# Engaging policy-makers, heath system managers, and policy analysts in the knowledge synthesis process: a scoping review

**DOI:** 10.1186/s13012-018-0717-x

**Published:** 2018-02-12

**Authors:** Andrea C. Tricco, Wasifa Zarin, Patricia Rios, Vera Nincic, Paul A. Khan, Marco Ghassemi, Sanober Diaz, Ba’ Pham, Sharon E. Straus, Etienne V. Langlois

**Affiliations:** 1 Li Ka Shing Knowledge Institute of St. Michael’s Hospital, 209 Victoria Street, Toronto, Ontario M5B 1T8 Canada; 2 Epidemiology Division, Dalla Lana School of Public Health, University of Toronto, 6th Floor, 155 College St, Toronto, Ontario M5T 3M7 Canada; 3 Department of Geriatric Medicine, Faculty of Medicine, University of Toronto, 27 King’s College Circle, Toronto, Ontario M5S 1A1 Canada; 4 Alliance for Health Policy and Systems Research, World Health Organization, Avenue Appia 20, 1211 Geneva, Switzerland

**Keywords:** Engagement, Knowledge user, Stakeholder, Knowledge translation, Health policy, Policy-relevant, Health system, Policy-maker, Knowledge synthesis

## Abstract

**Background:**

It is unclear how to engage a wide range of knowledge users in research. We aimed to map the evidence on engaging knowledge users with an emphasis on policy-makers, health system managers, and policy analysts in the knowledge synthesis process through a scoping review.

**Methods:**

We used the Joanna Briggs Institute guidance for scoping reviews. Nine electronic databases (e.g., MEDLINE), two grey literature sources (e.g., OpenSIGLE), and reference lists of relevant systematic reviews were searched from 1996 to August 2016. We included any type of study describing strategies, barriers and facilitators, or assessing the impact of engaging policy-makers, health system managers, and policy analysts in the knowledge synthesis process. Screening and data abstraction were conducted by two reviewers independently with a third reviewer resolving discrepancies. Frequency and thematic analyses were conducted.

**Results:**

After screening 8395 titles and abstracts followed by 394 full-texts, 84 unique documents and 7 companion reports fulfilled our eligibility criteria. All 84 documents were published in the last 10 years, and half were prepared in North America. The most common type of knowledge synthesis with knowledge user engagement was a systematic review (36%). The knowledge synthesis most commonly addressed an issue at the level of national healthcare system (48%) and focused on health services delivery (17%) in high-income countries (86%).

Policy-makers were the most common (64%) knowledge users, followed by healthcare professionals (49%) and government agencies as well as patients and caregivers (34%). Knowledge users were engaged in conceptualization and design (49%), literature search and data collection (52%), data synthesis and interpretation (71%), and knowledge dissemination and application (44%). Knowledge users were most commonly engaged as key informants through meetings and workshops as well as surveys, focus groups, and interviews either in-person or by telephone and emails. Knowledge user content expertise/awareness was a common facilitator (18%), while lack of time or opportunity to participate was a common barrier (12%).

**Conclusions:**

Knowledge users were most commonly engaged during the data synthesis and interpretation phases of the knowledge synthesis conduct. Researchers should document and evaluate knowledge user engagement in knowledge synthesis.

**Registration details:**

Open Science Framework (https://osf.io/4dy53/).

**Electronic supplementary material:**

The online version of this article (10.1186/s13012-018-0717-x) contains supplementary material, which is available to authorized users.

## Background

An estimated 85% of investment in health and biomedical research is wasted every year due to redundancies, failure to establish priorities based on needs of stakeholders (particularly end-users of knowledge), poorly designed research methods, and incomplete reporting of study results, leading to billions of dollars lost globally [1–3]. Stakeholders include those who are affected by, have an interest or stake in research [4], while knowledge users are subgroup of stakeholders who are likely to use research findings to make informed decisions about health systems and practices [5]. Knowledge users include but are not limited to patients and their informal caregivers or surrogate decision-makers (e.g., family, friends), healthcare providers (e.g., physicians, occupational therapists), policy-makers (e.g., Minister of Health, health officer), health system managers (e.g., hospital administrators, health unit managers), and policy analysts.

The overarching goal of knowledge user engagement in health research is to co-produce knowledge that is relevant and useful to those making real-world health decisions [6]. Early engagement of knowledge users in the research process may help establish research priorities and increase relevance of findings [7, 8]. To facilitate the use of research in decision-making, health systems and research funders are encouraging the engagement of knowledge users and other stakeholders in research [9].

Knowledge synthesis, such as a systematic review or a scoping review, is particularly useful for decision-makers because these research products provide a summary of the expansive evidence on a particular topic to inform decisions based on the totality of evidence [10, 11]. Co-production of evidence whereby researchers and knowledge users work together to conduct research increases the policy-relevance of research questions and fosters integration of findings into policy and practice [12–14]. However, the opportunities and approaches to engaging a wide range of knowledge users remain largely unexplored. Evidence is required to guide the process of engaging knowledge users in knowledge synthesis to identify engagement approaches that are effective, efficient, and meaningful. In addition, co-production of research by researchers and knowledge users requires additional time and funding, and it is imperative that the limited resources available for health research are used appropriately.

We undertook a scoping review to map the literature on engaging knowledge users in the knowledge synthesis process. Engagement of policy-makers, policy analysts, and health system managers were of particular interest, as these knowledge users are increasingly commissioning knowledge synthesis research products to meet their decision-making needs. The research questions (RQs) for our scoping review are provided below and outlined in our published protocol [15]:

(RQ1) In what context were policy-makers, health system managers, and policy analysts engaged (e.g., health system setting, high-income countries (HICs) versus low and middle-income countries (LMICs))?

(RQ2) What strategies exist to engage policy-makers, health system managers, and policy analysts in the knowledge synthesis process?

(RQ3) In studies describing strategies for engaging policy-makers, health system managers, and policy analysts, what outcomes do they measure to evaluate engagement mechanisms (e.g., attitudes, beliefs, knowledge) and what are the results (e.g. benefits, unintended consequences)?

(RQ4) What are the barriers and facilitators in engaging policy-makers, health system managers, and policy analysts in the knowledge synthesis process?

## Methods

### Commissioning agency

As part of a project to strengthen research capacity in LMICs, we were commissioned to conduct this scoping review by the Alliance for Health Policy and Systems Research (hereafter the Alliance), an international partnership hosted by the World Health Organization (WHO). We engaged with members of the Alliance throughout the review conduct.

### Study design

We selected the scoping review method [16] because we were interested in mapping the concepts relevant to engaging knowledge users in knowledge synthesis [16, 17]. The scoping review methodology is particularly useful when exploring an emerging and diverse knowledge-base, which makes the method well-matched to our RQs.

### Protocol

We drafted a scoping review protocol following the methods outlined by the Joanna Briggs Institute Methods Manual for scoping reviews [18] and reported findings using the elements provided in the Preferred Reporting Items for Systematic Reviews and Meta-analysis for Protocols (PRISMA-P) [19]. Our protocol was revised by the research team, registered with the Open Science Framework [20], and published in BMJ Open [15]. Since our full methods are available in our protocol, they are outlined briefly below.

### Eligibility criteria

Our eligibility criteria were conceptualized using the Population, Intervention, Comparator, Outcome, and Study design components [21], as follows:

#### Population

At minimum, the paper must mention at least one of the three knowledge user types specified in our RQs, which included policy-makers, policy analysts, and health system managers. Policy-makers are individuals at some level of government or decision-making institution, including but not limited to international organizations, non-governmental agencies or professional associations, who have responsibility for making recommendations to others [22]. Policy analysts are individuals at some level of government or decision-making institution, including but not limited to international organizations, non-governmental agencies or professional associations, responsible for analyzing data and informing decisions and recommendations [22]. Health system managers are individuals in a managerial or supervisory role in a health system with management or supervisory mandates, including implementers and public health officials [22].

#### Intervention

Papers that described any engagement strategy for policy-makers, health system managers, and policy analysts in the knowledge synthesis process were included. Engagement can be defined as “an iterative process of actively soliciting the knowledge, experience, judgment and values of individuals selected to represent a broad range of direct interests in a particular issue, for the dual purposes of: creating a shared understanding [and] making relevant, transparent and effective decisions” [8]. This scoping review limits knowledge user engagement to those opportunities that allow a meaningful interaction of the knowledge users in the research process from conception to design and completion and/or interpretation and uptake of results.

#### Comparators

Papers with or without a comparator group were eligible for inclusion.

#### Outcomes

Outcomes of interest were strategies, barriers, facilitators, and contextual factors for engaging health policy-makers, health system managers, or policy analysts in the conduct and use of knowledge synthesis. We also explored whether engagement strategies were evaluated regarding researcher and knowledge user attitudes, beliefs and knowledge of engagement as well as impact and effectiveness of engagement.

#### Study designs

We included any type of study design (e.g., qualitative or quantitative methods).

#### Time periods

To increase feasibility and timeliness of review completion, we restricted inclusion of the literature to the past 20 years.

#### Setting

All settings were eligible for inclusion.

#### Other

To increase feasibility and timeliness of review completion, only papers written in English were included.

Our full list of eligibility criteria can be found in Additional file 1: Appendix 1.

### Information sources and search strategy

The following electronic databases were searched by an experienced librarian (Dr. Jessie McGowan) from 1996 to August 15 2016: MEDLINE, Embase, ERIC, PsycINFO, Joanna Briggs, The Cochrane Library, EBM Reviews, The Campbell Library, and Social Work abstracts. The MEDLINE search strategy was peer-reviewed using the PRESS Statement [23] by a second librarian (Dr. Elise Cogo) and has been published in our protocol [15]. The main literature search was supplemented through searching GreyNet International [24] and OpenSIGLE [25] to locate unpublished (or grey) literature, such as conference abstracts and dissertations. All literature searches and full-text retrievals were executed by an experienced library technician (Ms. Alissa Epworth) and managed using Endnote [26]. Additionally, references from relevant review articles were scanned, and experts in the field were identified and contacted via email by the Alliance to identify additional sources of evidence.

### Study selection process

Literature search results were screened using our online Synthesi.SR software [27]. For level 1 screening of titles and abstracts, 3 pilot-tests were conducted on a total of 125 records. Once 80% agreement was achieved, pairs of reviewers (BP, MG, PR, PK, SD, VN, and WZ) independently screened remaining titles and abstracts. There were 403 (5%) discrepancies at level 1 screening, which were resolved by a third reviewer (WZ). For level 2 screening of potentially relevant full-text articles, 2 pilot-tests were conducted. When 70% agreement was achieved, pairs of reviewers (MG, PR, PK, SD, VN, and WZ) independently screened the full-text articles. There were 62 (18%) discrepancies at level 2 screening, which were resolved by a third reviewer (WZ).

### Data items and data abstraction process

We abstracted data on article characteristics (e.g., country of origin, funder), engagement characteristics and contextual factors (e.g., type of knowledge user, country income level [28], type of engagement activity, frequency and intensity of engagement, use of a framework [29] to inform the intervention), barriers and facilitators to engagement, and results of any formal assessment of engagement (e.g., attitudes, beliefs, knowledge, benefits, unintended consequences).

Data abstraction was conducted using a standardized Excel form that was developed *a priori* and pilot-tested on a sample of 5 included papers. After the team conducted 2 pilot-tests, data was abstracted by one reviewer and verified by another (MG, PK, SD, VN). Two experienced reviewers then quality checked the data for consistency and accuracy (WZ or PR).

### Risk of bias assessment

We did not conduct risk of bias assessment, which is consistent with the Joanna Briggs Institute Scoping Review Methods Manual [18] and scoping reviews on health-related topics [17].

### Synthesis of results

Results were synthesized using frequencies and thematic analysis [30]. Thematic analysis of open-text data was performed by one reviewer and verified by a second reviewer (PR, WZ). We used previously established nomenclature in our thematic analysis of barriers and facilitators to engaging knowledge users in health research [31, 32]. Engagement was coded and defined based on the framework established by Keown et al. [33]. Meta-analysis was not performed.

## Results

### Literature search

**Fig. 1.**
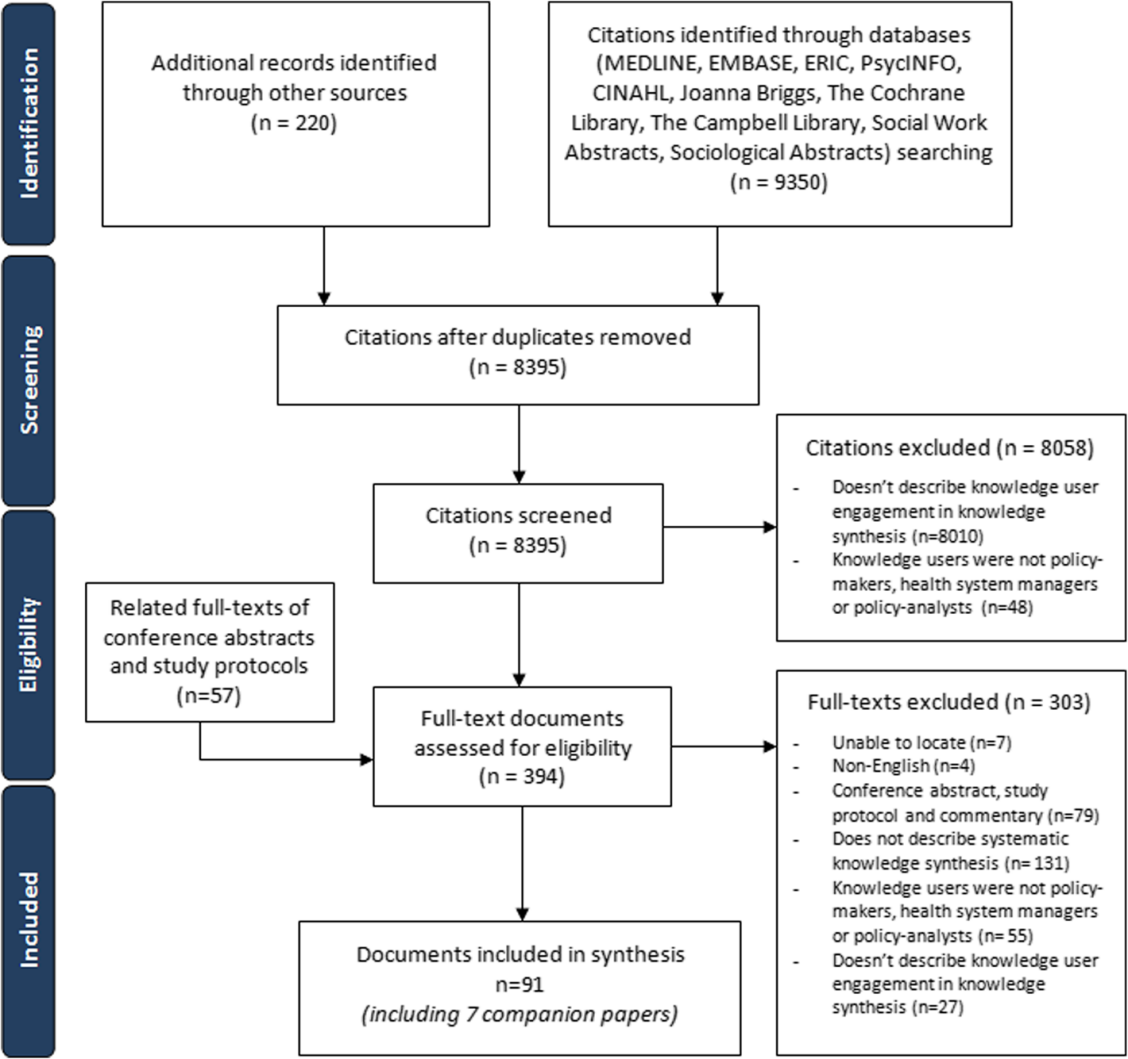
PRISMA flow diagram

After screening 8395 titles and abstracts and 394 full-text documents, 84 unique documents and 7 companion reports [34–40] (i.e., follow-up reports to the main documents included in our review) fulfilled our eligibility criteria (Fig. 1). The full citations can be found in Additional file 1: Appendix 2. Four documents were excluded because they were written in languages other than English, and 79 were excluded because they were conference abstracts, commentaries, or protocols that did not include relevant data. Six documents were identified through contacting experts in the field [11, 18, 24–27]. Six were unpublished reports, which were identified through our literature searches as well as through expert contact [41–46]. No relevant documents were identified through scanning reference lists.

### Characteristics of included documents (*n* = 84)

**Fig. 2.**
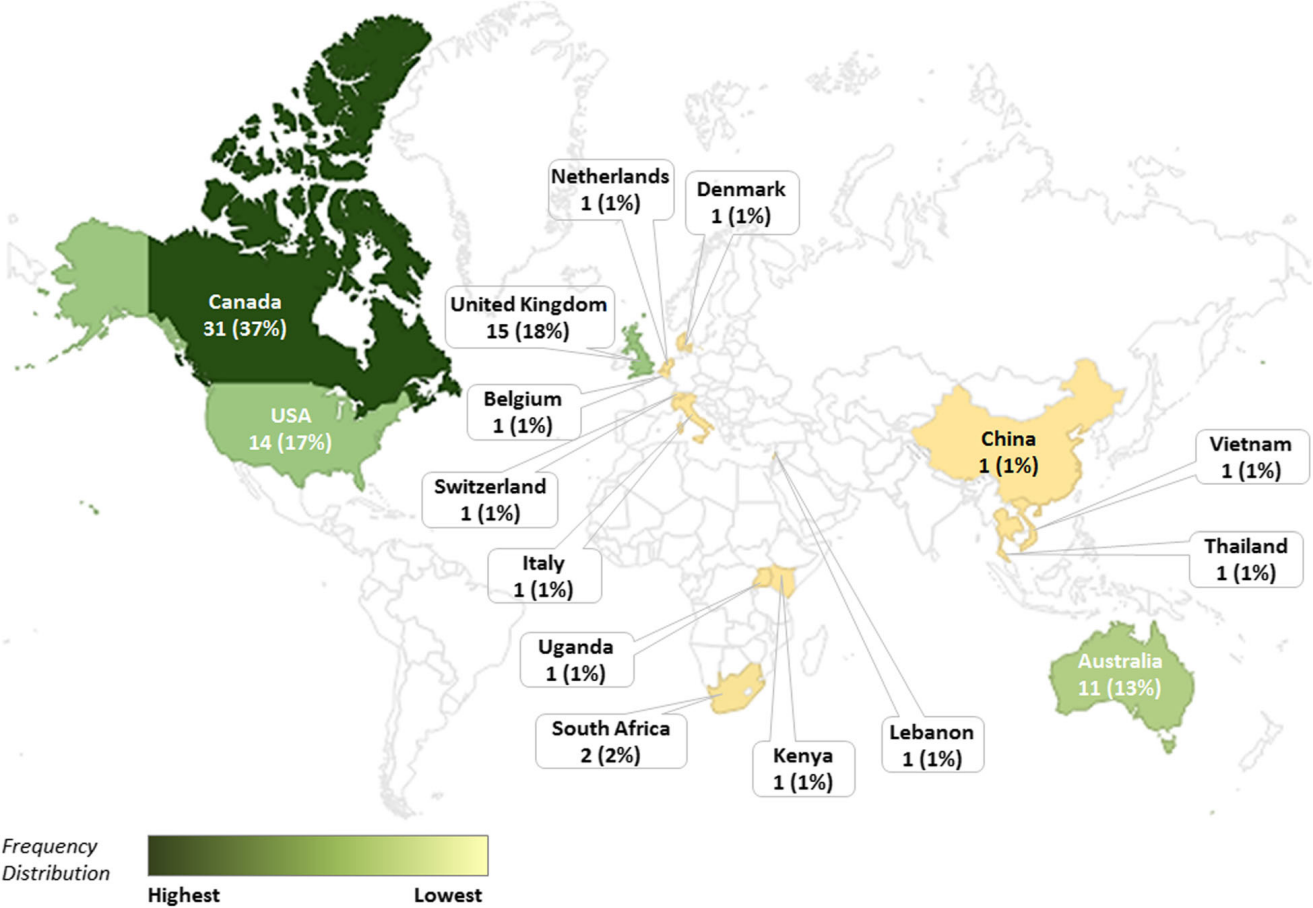
Choropleth of document distribution by geographic region

All documents were published in the last 10 years (Table 1) and originated predominantly in Canada (37%), USA (17%), UK (18%), and Australia (13%) (Fig. 2). The funding source was mainly public (79%), and Health Care Sciences & Services was the most common publishing journal discipline (31%). The types of documents were classified as application papers (87%) which are knowledge synthesis papers that described knowledge user engagement in the conduct of their research, descriptive papers (10%) which provided details of knowledge user engagement strategies developed by a research center or program, and methodology papers (4%) that studied knowledge user engagement in the knowledge synthesis process. The most common types of knowledge synthesis with knowledge user engagement were: systematic review (36%), literature review with a systematic literature search (19%), scoping review (14%), rapid review (12%), and realist review (6%) (Table 1).

**Table 1:** Document characteristics

Document characteristics (*n* = 84)		Count (%)
Year of publication	2005–2007	6 (7.1%)
	2008–2010	16 (19.0%)
	2011–2013	30 (35.7%)
	2014–2016	32 (38.1%)
Geographic region	Africa	4 (4.8%)
	Asia	4 (4.8%)
	Australia & New Zealand	11 (13.1%)
	Europe	20 (23.8%)
	North America	45 (53.6%)
Funding source type	Industry-sponsored	2 (2.4%)
	Non-sponsored	3 (3.6%)
	Not reported	13 (15.5%)
	Public-sponsored	66 (78.6%)
Journal discipline	General & Internal Medicine	4 (4.8%)
	Not applicable (reports)	6 (7.1%)
	Medicine, General & Internal	6 (7.1%)
	Health Policy & Services	7 (8.3%)
	Public, Environmental & Occupational Health	14 (16.7%)
	Other	21 (25.0%)
	Health Care Sciences & Services	26 (31.0%)
Knowledge synthesis method	Qualitative review	1 (1.2%)
	Critical Interpretive Synthesis	1 (1.2%)
	Mixed-method review	1 (1.2%)
	Health Technology Assessment	1 (1.2%)
	Scoping Review & Systematic Review	1 (1.2%)
	Horizontal scan	1 (1.2%)
	Rapid Realist Review	2 (2.4%)
	Overview of Reviews	3 (3.6%)
	Realist Review	5 (6.0%)
	Rapid Review	10 (11.9%)
	Scoping Review	12 (14.3%)
	Literature review	16 (19.0%)
	Systematic review	30 (35.7%)
Article type	Methodology paper	3 (3.6%)
	Descriptive paper	8 (9.5%)
	Application paper	73 (86.9%)

### RQ1: Contextual factors of included documents (*n* = 84)

The research most commonly addressed an issue at the level of the national healthcare systems (48%), followed by applied research settings (19%), and local healthcare systems (6%; Table 2). The knowledge synthesis product most commonly focused on health services delivery (17%), followed by knowledge translation (16%), and public health (10%). Most of the documents were produced in the context of high-income countries (86%), while 12% of the documents were in the context of low- and middle-income countries (see Table 3 for more details on LMICs).

**Table 2:** Contextual factors

Contextual factors (*n* = 84)		Count (%)
Settings	European Union Healthcare Systems	1 (1.2%)
	Community Health	2 (2.4%)
	National Public Health	2 (2.4%)
	Local hospital	2 (2.4%)
	Global health	3 (3.6%)
	Health network	4 (4.8%)
	Provincial/state healthcare system	4 (4.8%)
	Various policy settings	5 (6.0%)
	Local healthcare system	5 (6.0%)
	Applied research setting	16 (19.0%)
	National healthcare system	40 (47.6%)
Focus of knowledge synthesis	Health economics	1 (1.2%)
	Research reporting guideline	1 (1.2%)
	Health informatics	1 (1.2%)
	Emergency preparedness and management	1 (1.2%)
	Community engagement	1 (1.2%)
	Clinical practice guidelines	3 (3.6%)
	Medical intervention	3 (3.6%)
	Environmental/social determinants of health	3 (3.6%)
	Quality indicators	3 (3.6%)
	Health policy	4 (4.8%)
	Decision-aid tool	4 (4.8%)
	Research priority setting	5 (6.0%)
	Health human resources	8 (9.5%)
	Stakeholder engagement strategy in research	9 (10.7%)
	Public health	10 (11.9%)
	Knowledge translation	13 (15.5%)
	Health services delivery	14 (16.7%)
Country economy	High-income country	72 (85.7%)
	Low- and middle-income country	10 (11.9%)
	Middle and high income	1 (1.2%)
	Low, middle, and high income	1 (1.2%)

**Table 3:** Papers of knowledge user engagement in knowledge synthesis from LMICs

Author, year; country	Country income status, Context	Type of knowledge users involved	Type of engagement	Challenges to engagement	Benefits of engagement	Outcomes of engagement
Agweyu 2012 [68]; Kenya	Middle-income, National healthcare system	• Policy-makers	• Consultation with key informants • Formal meeting/workshop with expert panel	• Limited resources and an absence of mechanisms to rapidly gain wider opinions from key sources including patients, caregivers and policy-makers • Striking a balance between research evidence and expert opinions in the decision-making process	Not reported	Not reported
Akl 2016 [41]; Lebanon	Middle-income, Applied research settings	• Policy-makers	• Consultation with key informants • Focus groups, interviews and formal meeting/workshop with expert panel	Not reported	• A multidisciplinary team developed and validated the tool	Not reported
Buchan 2011 [69]; Brazil	Middle-income, National healthcare system	• Policy-makers	• Key informant interviews	• Time and resource limitations meant that only some of the key individuals who were informants on the issue could be consulted	• Key informants provided additional reports and grey literature for review and provided contextual evidence	Not reported
Clarke 2016 [70]; Cambodia	Middle-income, National healthcare system	• Healthcare professionals & organizations • Government agencies • Policy-makers	• Key informant interviews, focus groups and surveys	Not reported	Not reported	Not reported
Higashi 2011 [71]; Vietnam	Middle-income, Various policy settings	• Government agencies • Policy-makers	• Key informant interviews, focus groups and surveys • Formal meeting/workshop with key informant	Not reported	Not reported	Not reported
Muller 2005 [72]; South Africa	Middle-income, National healthcare system	• Government agencies • Non-government agencies • Patients, patient organizations & caregivers • Policy-makers	• Consultations with principal knowledge users • Working group	Not reported	Not reported	Not reported
Orem 2012 [73]; Uganda	Low-income, Various policy settings	• Policy-makers	• Key informant interviews	• Finding key informants with relevant interest in a given policy concern • Establishing unbiased opinion based on political interest	• Key informant interviews with policy-makers provided contextual considerations for knowledge synthesis and subsequent translation	Not reported
Sidibe 2014 [74]; Kenya	Middle-income, National healthcare system	• Community members & advocates • Funding bodies • Health system managers • Non-government agencies • Policy-makers	• Formal meeting/workshop with key informants	Not reported	Not reported	Not reported
Teerawattananon 2016 [75]; Thailand	Middle-income, National healthcare system	• Funding bodies • Healthcare professionals & organizations • Industry stakeholders • Non-government agencies • Patients, patient organizations & caregivers • Policy analysts • Policy-makers	• In-person Delphi with expert panel • Formal meeting/workshop with key informants	• Stakeholder topic expertise can limit the scope of the discussion	• Stakeholders can help prioritize research topics for assessment, help fine-tune research questions and the scope of study, and verify and validate preliminary results as well as fine-tune policy recommendations • Once final results have been obtained and policy recommendations have been formed, a stakeholder consultation meeting can help to verify and validate recommendations • Can be a powerful approach to systematically develop and legitimise policy-relevant HTA information	Not reported
Wiysonge 2012 [76]; South Africa	Middle-income, National healthcare system	• Health system managers	• Key informant interviews	Not reported	• Key informant interviews helped define the review scope and helped ensure the report addresses relevant practice issues	Not reported

### RQ2: Methodology documents (*n* = 3)

One methodology document conducted 18 key informant interviews with policy-makers and systematic review producers to identify institutional mechanisms to increase demand for and facilitate conduct of policy-relevant systematic reviews [47]. The authors proposed four models for achieving policy-relevant systematic reviews with an emphasis on policy-maker engagement based on knowledge user needs and timelines as well as complexity of the research question. The authors concluded that early engagement with managers and policy-makers can improve clarity and consensus of definitions and maximize relevance of systematic reviews.

Another methodology document conducted a systematic literature search of stakeholder engagement and 13 key informant interviews on prioritizing research [48]. The authors included 56 papers that used mixed qualitative/quantitative approaches to engaging stakeholders using in-person, online, or teleconference modalities. Prioritization of research was often achieved using structured ranking or Delphi methods. Ten factors for successful engagement were recommended and are outlined in Table 4.

**Table 4:** Methodology papers of knowledge user engagement in knowledge synthesis

Article, Year; Country	Country income status, Context	Information source	Type of engagement	Challenges to engagement	Factors for successful engagements
Cottrell 2014 [46]; USA	High-income, Applied research settings	24 articles, 34 Key informant interviews	Wide variety	• Additional time and resources • Selection of stakeholders/achieve representativeness • Reliability/consistency in participation • Maintain confidentiality • Manage and support stakeholdersOvercome tokenism	• Engage stakeholders early in the process to establish credibility • Anticipate controversies in stakeholder opinions • Ensure transparency and accountability
Guise 2013 [48]; USA	Low, middle and high income, Applied research settings	56 articles, 13 Key informant interviews	• One-on-one interviews • Focus groups • Citizen juries • Town meetings • Workshops/symposia/conferences • Ranking and Delphi/Nominal group techniques	• Lack of time on the part of stakeholders (busy) • Lack of release time and compensation for members of the public • Researcher need for quick response (time frame too short for community to weigh in) • Stakeholder needs not met in previous engagement	• Engage stakeholders early in the process • Clearly detail expectations (e.g., timelines, tasks) • Maintain ongoing relationships to building trust and credibility • Provide opportunities for people to ask questions before meetings • Provide pre-meeting information materials • Pre-meeting “icebreakers,” especially when engaging stakeholders with differing experiences/perspectives • Include someone with similar training as the stakeholder can be helpful • Respect and welcome all stakeholder opinions • Follow-up presentation of results is important to stakeholders • Be clear about the stakeholder roles, do not expect community members to do academic duties • Be sensitive to the time constraints of all stakeholders
Oliver 2016 [47]; UK	High-income, Various policy settings	18 Key informant interviews	• Knowledge broker to facilitate conversations • Advisory panel and Expert panel for consultations	• Lack of knowledge and understanding between researchers and policy-makers • Considerable time required to negotiate review questions with policy-makers • Researchers lacking experience with stakeholder engagementIdentifying appropriate stakeholder to engage • Managing timelines, resources and costs associated with engagement	• Engage stakeholders early in the process • Manage stakeholder expectations • Maintain appropriate communication and transparency • Face-to-face meetings were more successful than telephone calls (not formally evaluated)

A third methodology document examined the benefits of engaging a range of stakeholders in systematic reviews through a review of 24 papers and 34 key informant interviews [46]. The authors noted that although a number of benefits and challenges to engaging stakeholders were identified, none of the studies formally evaluated engagement.

### RQ2: Descriptive documents (*n* = 8)

The eight descriptive documents [33, 49–54] described engagement approaches used for the following: Healthcare Improvement Scotland [53], Greater London Authority (UK) [55], Agency for Healthcare Research and Quality (US) [49], Samueli Institute (US) [51], Institute for Work and Health (Canada), Ottawa Hospital Research Institute (Canada) [52], and various funding and governmental agencies within Canada [33, 50, 54]. Most of the approaches involved consultations. One paper described [33] engagement of knowledge users as part of the review team. Further results can be found in Table 5.

**Table 5:** Descriptive papers of knowledge user engagement in knowledge synthesis

Author, Year, Country	Country income group, Context	Type of knowledge users involved	Type of engagement	Challenges to engagement	Benefits of engagement	Outcomes of engagement
Atkins 2005 [49], USA	High-income, National healthcare system	• Policy-makers	• Consultation with expert panel	• Establishing early buy-in • Managing knowledge user expectation what evidence is available and what questions can be answered	• Early involvement in the research process can ensure the report addresses relevant clinical or policy issues • Knowing how the knowledge user will use the findings of the report can help inform data synthesis	Not reported
Best 2009 [50], Canada	High-income, Various levels of government decision-makers	• Policy-makers	• Consultation with principal knowledge users and expert panel	Not reported	Not reported	Participating content experts and decision-makers have been highly satisfied. (Not formally evaluated)
Crawford 2015 [51], USA	High-income, Various levels of government decision-makers	• Healthcare professionals & organizations • Patients, patient organizations & caregivers • Policy-makers	• Consultation with Steering group	Not reported	• Involving stakeholders ensures research focus stays relevant to the end-user • Allows for translation from research to practice to occur more effectively	Not reported
Keown 2008 [33], Canada	High-income, Various levels of government decision-makers	• Community members & advocates • Government agencies • Policy-makers • Regulatory bodies	• Consultation with key informants throughout he review • Principal knowledge users included as a review team member	• Balancing methodological rigor with flexibility to stakeholder needs • Stakeholder interactions can be time and resource-intensive • Some stakeholder feedback may not be feasible due to time and resource limitations • Finding an appropriate and knowledgeable stakeholder to participate as a review team member can be difficult	• Stakeholders’ input added depth to the review • Timing of stakeholder participation leads to specific advantages (e.g., early engagement led to exhaustive literature search and refined research questions, later engagement helped refine the report) • Research findings are more useful and relevant to end-users • Opportunity to build capacity of the knowledge users in research methods • On-going collaboration increases the chances for future collaborations	The stakeholder engagement experience has been positive (not formally evaluated)
Khangura 2012 [52], Canada	High-income, Local healthcare system	• Health system managers • Policy-makers	• Consultation with principal knowledge users	Not reported	Not reported	Not reported
McIntosh 2016 [53], UK	High-income, National healthcare system	• Government agencies • Healthcare professionals & organizations • Industry stakeholder • Patients, patient organizations & caregivers	• Consultation with principal knowledge users and expert panel • Formal meeting/ workshop with advisory group, principal knowledge users and expert panel	• Requires development of efficient and flexible methods to identify and engage appropriate contributors • Managing expectations in order to ensure that rapid review conclusionsare not oriented by vested interests • Striking the right balance between engagement and pragmatism	Not reported	Upon completion, action review methods are used to solicit feedback from the topic referrer on whether review met expectations and what impact the evidence review and advice had. Surveys and semi-structured interviews conducted to explore perceptions of the utility and impact of rapid review-based advice among key decision-makers, including directors of finance, planning, public health and medicine
Mindell 2010 [55], UK	High-income, Local healthcare system	• Government agencies • Policy-makers	• Consultation with steering group • Formal meeting/ workshop with key informants	Not reported	Not reported	Not reported
Saul 2013 [54], Canada	High-income, Various levels of government decision-makers	• Policy-makers	• Consultation with principal knowledge users	• Maintaining on-going membership and engagement in rapidly changing political environments where membership of the advisory group may change during the course of a given project	• Advisory group role allows key agency or government staff to be engaged in the process without requiring excessive time commitments	Not reported

### RQ2: Application papers (*n* = 73)

**Fig. 3.**
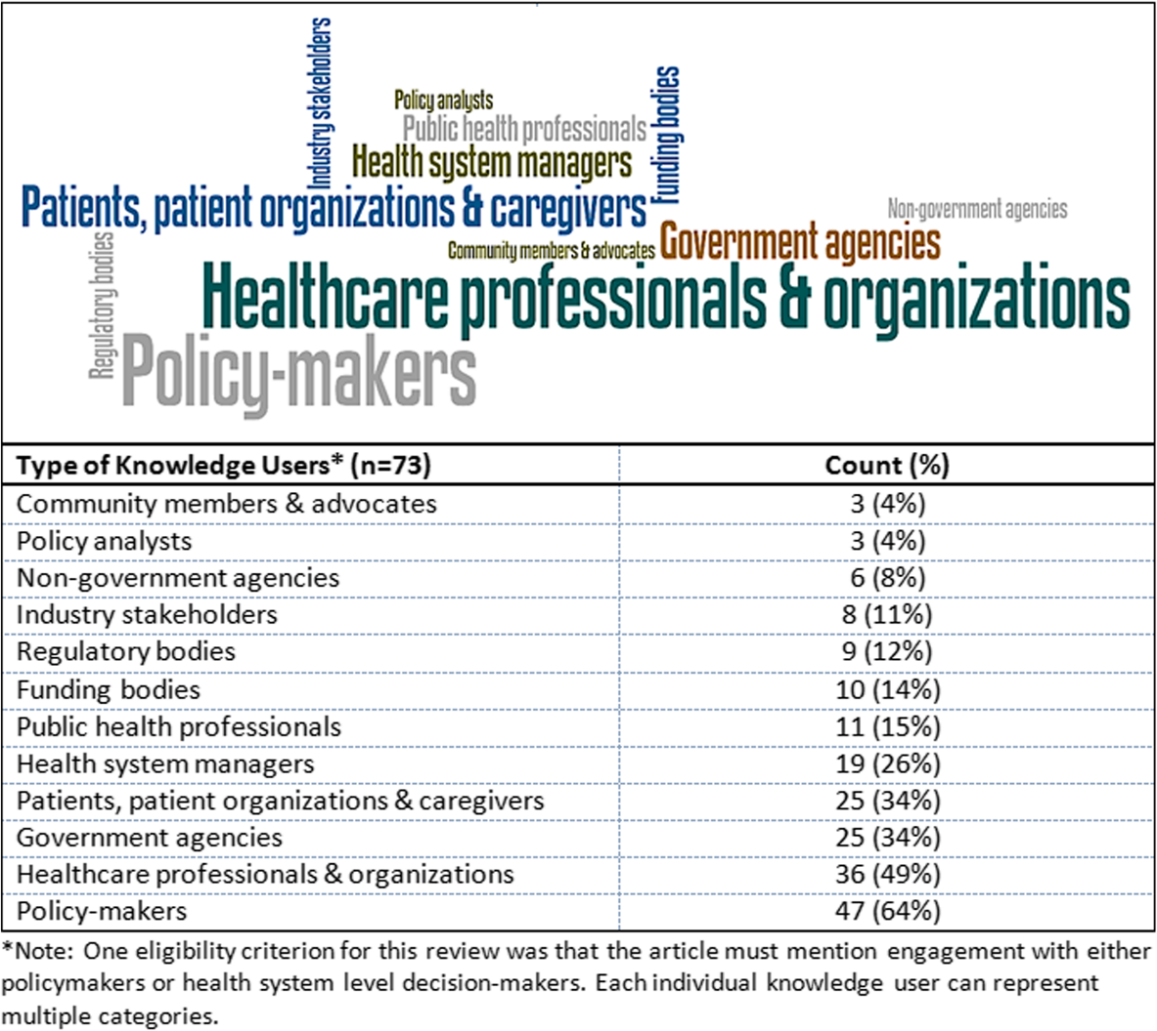
Types of knowledge users

Seventy-three knowledge synthesis documents reported knowledge user engagement in the research process. Policy-makers were the most common (64%) type of knowledge users to be engaged in the knowledge synthesis process, followed by healthcare professionals and organizations (49%), and government agencies as well as patient organizations and caregivers (34%; Fig. 3).

**Fig. 4.**
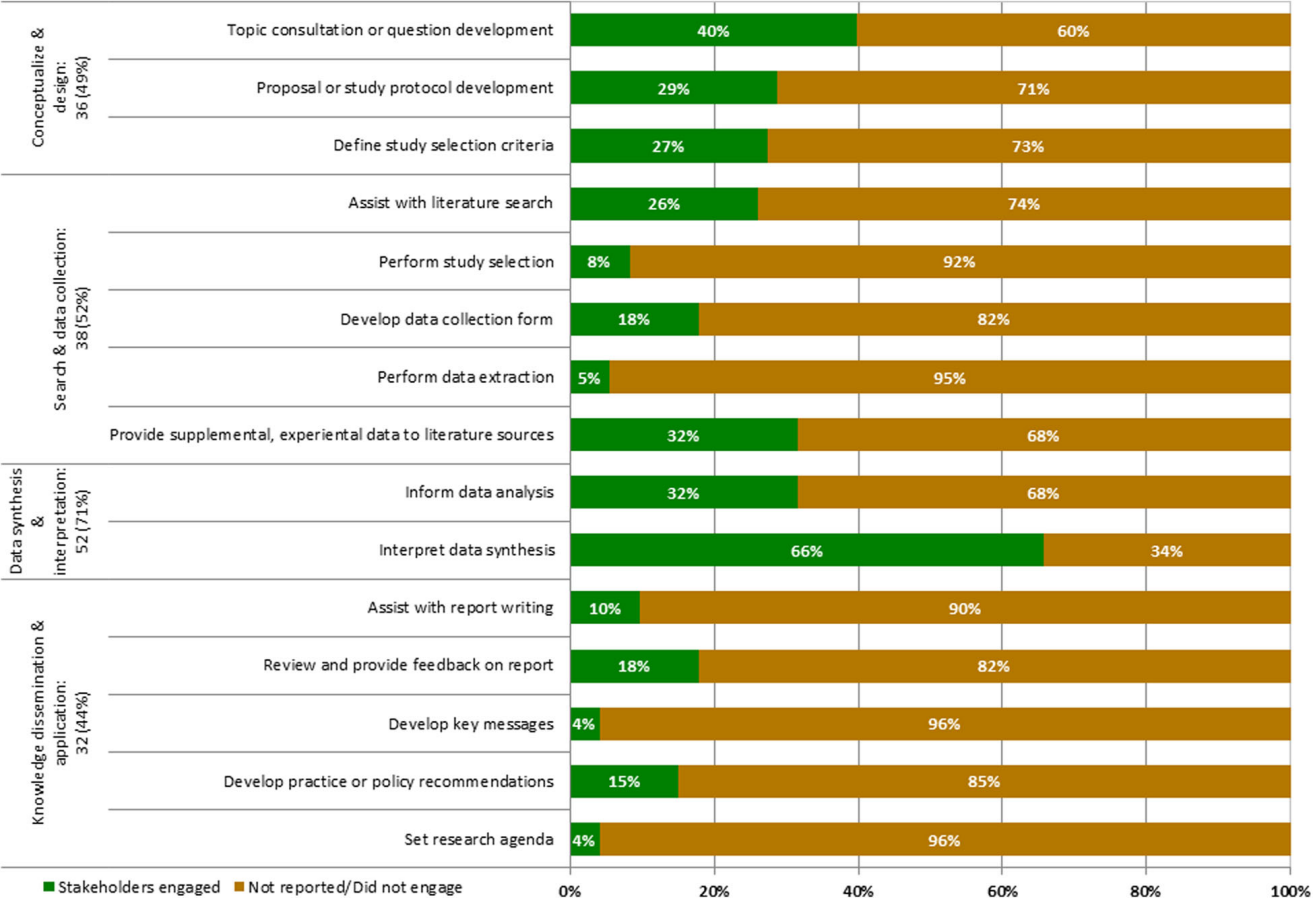
Distribution of knowledge user engagement by steps in the knowledge synthesis process

The points of engagement in the knowledge synthesis process occurred at the onset of the review to conceptualize and plan the research (49%), where knowledge users were engaged to either select the research topic or refine research questions (40%), develop the study proposal or protocol (29%) or define study selection criteria (27%) (Fig. 4). Knowledge users were also involved at the literature search or data collection phase (52%) to either assist with the literature search (26%), help with study selection (8%), provide input on the data collection form (18%), help with data collection (5%) or provide experiential data to supplement the data obtained from the literature searches (32%). At the data synthesis and interpretation stage (71%), knowledge users informed data analysis (32%) or helped interpret the results (66%). During the knowledge dissemination and application phase (44%), knowledge users assisted with the report writing (10%), reviewed and provided feedback on the draft report (18%), helped develop key messages (4%), developed practice or policy recommendations (15%), or established the future research agenda (4%).

**Fig. 5.**
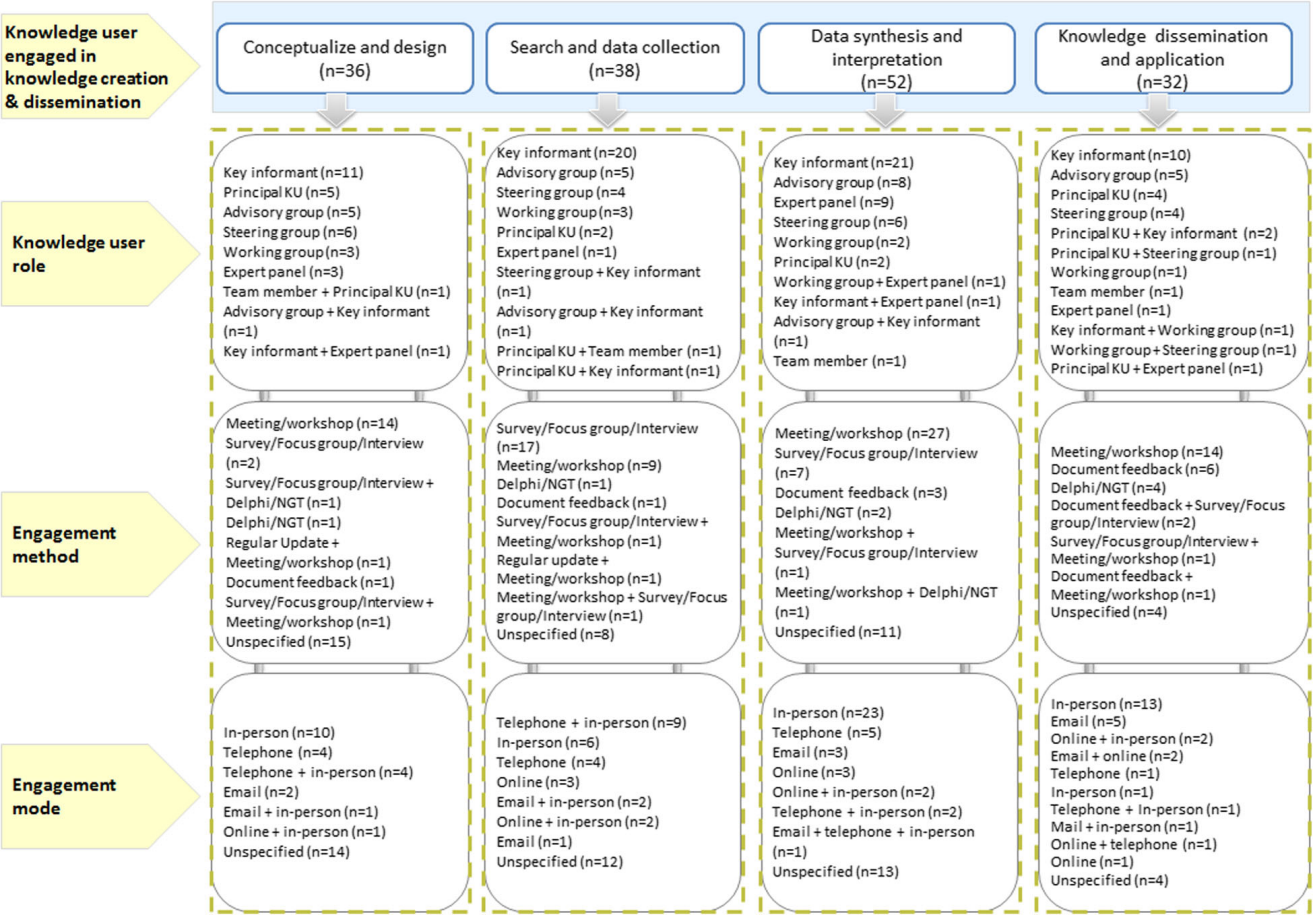
Engagement strategy framework

Knowledge users were most commonly engaged as key informants across the four stages of the knowledge synthesis process (Fig. 5). Other roles included advisors (i.e., knowledge users provide high-level recommendations and advice on the design and method and is typically engaged at various stages of a review), expert panel (i.e., knowledge users provide specialized input/opinion on the topic and is typically engaged at a specific stage of a review), steering group (i.e., knowledge users provide strategic decisions on the direction of the research project and is consulted at various stages of a review), or as a team member (i.e., knowledge user is included as part of the review team). Full definitions for all terms can be found in Additional file 1: Appendix 3.

Frequently used methods of engagement were structured meetings or workshops and information gathering by means of surveys, focus groups or interviews across all four stages. Other methods of engagement included nominal group techniques or Delphi approaches to problem-solve and reach decisions in a group setting as well as circulating documents for feedback, and sending regular updates to relevant knowledge users. Knowledge users were most commonly engaged in-person or telephone across all four stages of a knowledge synthesis. Other forums for engagement included online platforms and email discussions.

**Fig. 6.**
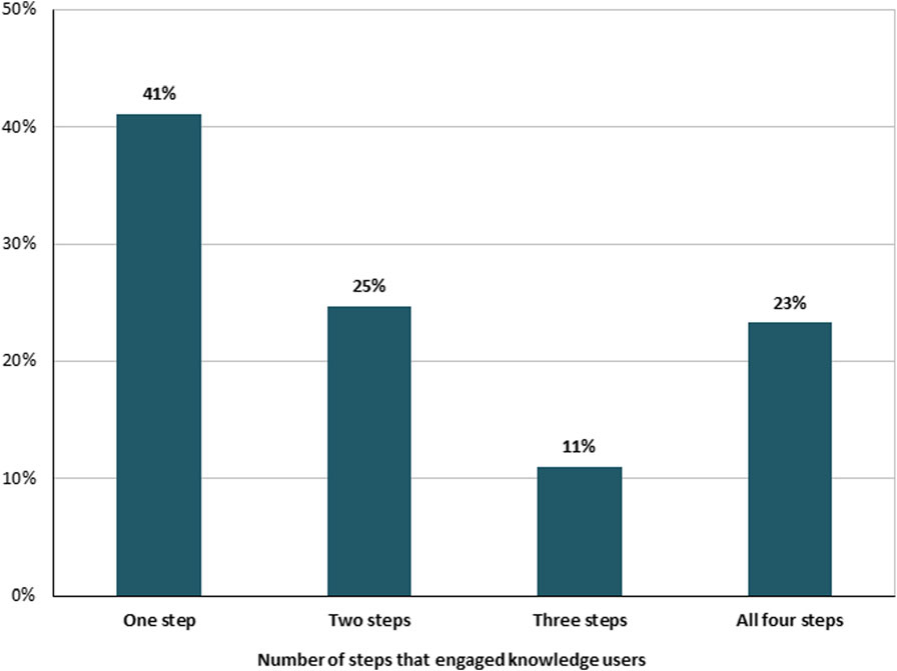
Frequency of engagement

The frequency of knowledge user engagement varied across the 73 application documents (Fig. 6). Knowledge users were engaged only once during the knowledge synthesis process in two-fifths of the documents, twice in one-quarter of the documents, three times in one-tenth of the documents and in all four stages in nearly one-quarter of the documents.

### RQ2: Frameworks used to inform engagement strategy

One document reported the use of a framework for engagement in research [56] called the 7Ps of Stakeholder Engagement and Six Stages of Research [57]. The 7Ps are (1) patients and the public, (2) providers, (3) purchasers, (4) payers, (5) policy-makers, (6) product makers, and (7) principal investigators. The six stages of research are (1) evidence prioritization, (2) evidence generation, (3) evidence synthesis, (4) evidence integration, (5) dissemination and application, and (6) feedback and assessment [57]. The authors of this framework recommended the following: prioritizing engagement through funding opportunities and other initiatives and adopting a common taxonomy when working with knowledge users, experimenting with different engagement strategies and evaluating them on an ongoing basis, and reporting outcomes and continuous quality improvement to iterate and implement changes when required.

Another document provided a conceptual framework on the models and mechanisms for engaging policy-makers in systematic reviews that focus on health policy and systems research [47]. Mechanisms that can be used to bolster engagement with policy-makers included the following: finding ongoing funding so researchers can answer questions posed by policy-makers, providing capacity-building to researchers and policy-makers to support engagement, and having team members with experience working closely with policy-makers.

### RQ3: Outcomes of engagement (*n* = 84)

None of the included documents conducted a formal evaluation of engagement; measurement tools specific to engagement were not identified. The authors of one paper asked participating knowledge users to answer an anonymous survey and 100% reported that the information provided in the review was “very” or “somewhat” useful in their decision-making [58]. One study [46] suggested ways to measure engagement in future research, including tracking how the research question, eligibility criteria, or other aspects of the review were modified after engagement, comparing reviews on the same topic with engagement and without engagement, retrospectively evaluating reviews that were conducted without engagement to determine their impact, or deliberately phasing in engagement at different parts of the process to measure how the engagement impacted the review.

### RQ4: Barriers and facilitators to engagement (*n* = 31)

Thirty-one documents reported on 16 factors that were considered barriers or facilitators to engagement (Table 6). The most common facilitators were content expertise/awareness of the knowledge user (17%), establishing partnership with knowledge users early in the research process (8%), and having forums for ongoing interaction (7%). The most common barriers reported were lack of time or opportunity for engagement (11%) and when knowledge users lacked expertise/awareness of the topic (content) (6%).

**Table 6:** Barriers and facilitators to engagement

Factors reported in 31 papers	Seen as a facilitator	Seen as a barrier
	Count (%)	Count (%)
Available resources (e.g., personnel, material)	0 (0.0%)	2 (2.4%)
Capacity and established methods for engagement	3 (3.6%)	1 (1.2%)
Clear expectations and responsibilities	3 (3.6%)	1 (1.2%)
Contact with knowledge users	2 (2.4%)	1 (1.2%)
Differing values	1 (1.2%)	0 (0.0%)
Establishment of partnership early in the research process	7 (8.3%)	0 (0.0%)
Establishment of unbiased consensus	1 (1.2%)	2 (2.4%)
Forums for interaction	6 (7.1%)	1 (1.2%)
Geographic distance	0 (0.0%)	1 (1.2%)
Ongoing collaboration with partners	4 (4.8%)	0 (0.0%)
Knowledge user research skills	1 (1.2%)	1 (1.2%)
Knowledge user topic expertise/awareness	*14 (16.7%)*	5 (6.0%)
Relationship with knowledge users	2 (2.4%)	0 (0.0%)
Timing and opportunity	3 (3.6%)	*9 (10.7%)*
Training/mentoring of researchers and knowledge users	2 (2.4%)	0 (0.0%)
Willingness to participate	1 (1.2%)	2 (2.4%)

## Discussion

The included documents were predominantly conducted at the level of a national healthcare system and focused on health services delivery in the context of high-income countries. We did not identify any distinguishing trends in engagement when we compared knowledge user engagement across country income groups and other contextual factors. We did not identify differences in results over time or across settings, for phases of engagement, or how the engagement was conducted. This might be because the practice of engaging knowledge users in knowledge synthesis is still relatively new.

Knowledge users were most commonly engaged as key informants who were engaged through structured meetings or workshops and surveys, focus groups or interviews. Knowledge users were engaged only once during the knowledge synthesis process in two-fifths of the documents, twice in one-quarter of the documents, three times in one-tenth of the documents, and across all four stages in nearly one-quarter of the documents. None of the documents conducted a formal evaluation of engagement and measurement tools specific to engagement were not identified. Sixteen barriers and facilitators were identified. The most common facilitator was content expertise/awareness of the knowledge user, whereas the most common barrier was lack of time or opportunity for engagement.

There are numerous perceived benefits to engaging policy-makers, policy analysts, and health system managers in knowledge synthesis. Examples include more comprehensive literature searches, improved rigor of knowledge synthesis findings, greater clarity of results [59] as well as greater relevance, uptake, and usefulness of results. However, the results of our scoping review suggest that very little research has been conducted in this area. The research that has been conducted is purely descriptive in nature and a formal evaluation of engagement approaches and outcomes was not identified. A future study could evaluate engagement using a variety of methods, such as documenting how the knowledge synthesis process and results were modified after engagement or testing engagement at different points of the knowledge synthesis process to see how engagement influences research impact.

We identified several factors that may enhance engagement of knowledge users in knowledge synthesis process that are within the researcher’s control, for example, engaging knowledge users before the synthesis begins; clearly outlining expectations regarding stakeholder’s role and time commitment; identifying funding opportunities to work closely with policy-makers; providing time for question and answer opportunities; conducting ice breaker activities; providing materials in advance of meetings; considering knowledge user comments as being equal to those received from researchers; being sensitive to knowledge user’s time; presenting results to knowledge users; and using a neutral facilitator. As none of these have been formally evaluated, we cannot comment on the effectiveness of any of these approaches. As such, the type and intensity of engagement should be meaningful and tailored to available resources, including time and funding. To better define knowledge user engagement in knowledge synthesis, researchers should discretely identify the desired benefits and impacts and effectiveness of engagement and develop systematic and reproducible methods and indicators for formal evaluation.

There were four main phases when engagement took place, including conception and design of research, search and data collection, data synthesis and interpretation, and knowledge dissemination and application.

Knowledge users were most often engaged as key informants across the four stages of the knowledge synthesis process to obtain advice, feedback, and opinions. However, there is increasing interest globally in co-design and co-development of research with knowledge users and using research to inform public policy [60, 61]. Co-creation of science is gaining momentum to integrate research and decision-making cycles and incorporate knowledge generation in complex policy planning and implementation, ultimately enhancing the usability and impact of research [12, 62, 63]. It will be important to test the utility of the co-design and co-creation of knowledge synthesis in the future.

Two conceptual frameworks were identified that provided a structure and mechanism to facilitate knowledge user engagement in knowledge synthesis. These were the 7Ps of Stakeholder Engagement and Six Stages of Research framework [58] and a conceptual framework on the models and mechanisms for engaging policy-makers in systematic reviews that focus on health policy and systems research [47]. An additional framework can also be used: the online survey patient and public engagement questionnaire (PPEQ) [64]. Members of our research team are currently conducting a study to test the level of engagement of knowledge users in a systematic review using the PPEQ [65], which will provide clarity to the field.

Two additional frameworks [66, 67] to engage stakeholders in knowledge synthesis were published after the literature search date and completion of our scoping review. Haddaway and colleagues [66] discussed a framework including approaches for engaging stakeholders during systematic reviews in the field of environmental management. Land and colleagues [67] described an empirically tested five-step approach for stakeholder engagement in prioritization and planning of environmental evidence syntheses that the Mistra Council for Evidence-based Environmental Management has been using. These frameworks may also be of relevance to knowledge synthesis within health and should be examined more closely in the future.

The strengths of our scoping review include a comprehensive literature search of multiple electronic databases as well as unpublished sources. We also followed the rigorous scoping review methods suggested by the Joanna Briggs Institute. We engaged with the principal knowledge user (EVL) throughout the review process who provided input in our research questions, review protocol, eligibility criteria of papers, reviewed this manuscript, and helped interpret our findings. In terms of dissemination plans, in addition to publication of this manuscript, we will prepare a1-page policy brief which will be made available on our website (https://knowledgetranslation.net/) and present at international conferences. Team members will also use their networks to encourage broad dissemination of results.

There are limitations to our scoping review process. To increase feasibility, we limited inclusion to documents made available in the past 20 years. However, this is likely not a substantial limitation, as all of the included documents were made available in the past 10 years. We also limited inclusion to studies written in English, which may have resulted in the exclusion of eligible studies from LMIC settings for RQ1. Given the large number of documents included, the data were abstracted by one reviewer and verified by a second reviewer. However, the data are likely valid, as a pilot-test was conducted prior to embarking on data abstraction with the entire team and a second reviewer who is an experienced research coordinator on the team verified all data. Often the included documents did not distinguish between stakeholders (i.e., those who are affected by or have an interest or stake in research [4]) and knowledge users (i.e., a subgroup of stakeholders who are likely to use research findings to make informed decisions about health systems and practices [5]), which is likely due to inconsistent use of the terms in the literature. As such, our results are likely applicable to both stakeholder and knowledge user participants. Furthermore, the reporting of knowledge user engagement methods varied considerably in their completeness across the literature, and as such, our data are limited by the details described in the literature. For example, most papers described steps to engage knowledge users but did not provide details on non-response or unsuccessful engagement.

## Conclusions

Engaging policy-makers, policy analysts, and health system managers in knowledge synthesis usually occurs at the beginning or end of the knowledge synthesis process. However, ongoing engagement throughout the review process may lead to more relevant and user-friendly results. The type and intensity of engagement should be meaningful and tailored to available resources, including time and funding. Researchers should document and evaluate engagement activities in knowledge synthesis on an ongoing basis. It is important to document and test knowledge user engagement in knowledge synthesis in the future, to advance the field.

## Additional files

Additional file 1: Appendices. The appendices includes all supplemental information. Appendix 1. Inclusion and exclusion criteria. Appendix 2. List of included papers. Appendix 3. Definitions. (DOCX 37 kb)

## Abbreviations

HICs: High-income countries
LMICs: Low- and middle-income countries
PPEQ: Patient and public engagement questionnaire
PRISMA-P: Preferred reporting items for systematic reviews and meta-analysis for protocols
RQ: Research questions
WHO: World Health Organization
