# Correction to: Engaging policy-makers, health system managers, and policy analysts in the knowledge synthesis process: a scoping review

**DOI:** 10.1186/s13012-018-0749-2

**Published:** 2018-04-16

**Authors:** Andrea C. Tricco, Wasifa Zarin, Patricia Rios, Vera Nincic, Paul A. Khan, Marco Ghassemi, Sanober Diaz, Ba’ Pham, Sharon E. Straus, Etienne V. Langlois

**Affiliations:** 1 Li Ka Shing Knowledge Institute of St. Michael’s Hospital, 209 Victoria Street, Toronto, Ontario M5B 1T8 Canada; 2 Epidemiology Division, Dalla Lana School of Public Health, University of Toronto, 6th Floor, 155 College St, Toronto, Ontario M5T 3M7 Canada; 3 Department of Geriatric Medicine, Faculty of Medicine, University of Toronto, 27 King’s College Circle, Toronto, Ontario M5S 1A1 Canada; 4 Alliance for Health Policy and Systems Research, World Health Organization, Avenue Appia 20, 1211 Geneva, Switzerland

## Correction

Following the publication of the original article [1], it was brought to our attention that the letter ‘l’ was unfortunately omitted from the word ‘health’ in the article’s title.

The correct title should read: ‘Engaging policy-makers, health system managers, and policy analysts in the knowledge synthesis process: a scoping review’.

The correct title has been included in this Correction and updated in the original article.
