# Supplementary material for: Engaging policy-makers, heath system managers, and policy analysts in the knowledge synthesis process: a scoping review
Source: Implement Sci. 2018 Feb 12;13:31. doi: 10.1186/s13012-018-0717-x (PMC5809959; doi:10.1186/s13012-018-0717-x)
Supplement: Supplementary file 1 — Appendices. The appendices includes all supplemental information. Appendix 1. Inclusion and exclusion criteria. Appendix 2. List of included papers. Appendix 3. Definitions. (DOCX 37 kb) [file 13012_2018_717_MOESM1_ESM.docx]

Additional File 1

[**Appendix 1.** Inclusion and exclusion criteria 2](#_Toc493768973)

[**Appendix 2.** List of included papers 4](#_Toc493768974)

[**Appendix 3.** Definitions 10](#_Toc493768975)

# **Appendix 1.** Inclusion and exclusion criteria

| **Inclusion criteria** | **Exclusion criteria** |
| --- | --- |
| *Population* | |
| Knowledge users who are likely to use research findings to make informed decisions about health policies, programs and/or practices.  Must include at least one knowledge user from the following groups:   - Policy-makers - Health system managers - Policy analysts   Other knowledge users may include:   - Patient, patient organizations, caregivers - Funding bodies - Industry - Healthcare institutions and organizations - Healthcare professionals and professional colleges/associations - Community members and advocates | Exclude if paper does not include at least one knowledge-user who is a policy-maker, health system manager or policy analyst.  Exclude if paper only included knowledge-users from the other categories.  Exclude if paper only included researchers, academics and content experts. |
| *Interventions* | |
| Knowledge users actively consulted for solicited input at least once in the research process, including:   - Planning phase - Conduct of review - Development of knowledge tools/products - Dissemination and implementation phase | Knowledge users were only passively engaged, typically at the end of the research process   - Presenting research findings at a conference without any discussion or feedback from knowledge users - Circulating final report or published manuscript to potential knowledge users without any opportunity for feedback from knowledge users - Disseminating various knowledge tools/products to knowledge users without any opportunity for feedback/input in the development |
| *Comparators* | |
| Include papers with or without a comparator group | Not applicable; all comparators are eligible, including papers without a comparator group. |
| *Outcomes* | |
| Must report at least one of the following:   - Engagement methods - Barriers or facilitators to engaging knowledge users - Formal evaluation of effectiveness and shortcomings of knowledge user engagement in the research process | No relevant information provided. |
| *Study design* | |
| Any study design including:   - Knowledge synthesis with systematic literature search (e.g., systematic reviews, rapid reviews, scoping reviews) - Case reports or series - Experimental or observational studies | Not applicable. |

# **Appendix 2.** List of included papers

| **Main documents (n=84)** |
| --- |
| 1. Committee on Integrating Primary Care and Public Health. Primary Care and Public Health-Exploring Integration to Improve Population Health 2012.  2. Adam-Poupart A, Labreche F, Smargiassi A, Duguay P, Busque MA, Gagne C, et al. Climate change and Occupational Health and Safety in a temperate climate: potential impacts and research priorities in Quebec, Canada. Industrial health. 2013;51(1):68-78.  3. Addington DE, McKenzie E, Wang J, Smith HP, Adams B, Ismail Z. Development of a core set of performance measures for evaluating schizophrenia treatment services. Psychiatric services (Washington, DC). 2012;63(6):584-91.  4. Agweyu A, Opiyo N, English M. Experience developing national evidence-based clinical guidelines for childhood pneumonia in a low-income setting--making the GRADE? BMC pediatrics. 2012;12:1.  5. Akl E, Fadlallah R, Ghandour L, Kdouh O, Langlois E, Lavis J, et al. The SPARK Tool for Prioritizing Questions for Systematic Reviews in Health Policy and Systems Research: Development and initial Validation. Health Research Policy Systems.15(1):77.  6. Archambault PM, van de Belt TH, Grajales FJ, 3rd, Faber MJ, Kuziemsky CE, Gagnon S, et al. Wikis and collaborative writing applications in health care: a scoping review. Journal of medical Internet research. 2013;15(10):e210.  7. Atkins D, Fink K, Slutsky J. Better information for better health care: the Evidence-based Practice Center program and the Agency for Healthcare Research and Quality. Annals of internal medicine. 2005;142(12 Pt 2):1035-41.  8. Bennett K, Rhodes AE, Duda S, Cheung AH, Manassis K, Links P, et al. A Youth Suicide Prevention Plan for Canada: A Systematic Review of Reviews. Canadian journal of psychiatry Revue canadienne de psychiatrie. 2015;60(6):245-57.  9. Bernatsky S, Lix L, O'Donnell S, Lacaille D. Consensus statements for the use of administrative health data in rheumatic disease research and surveillance. The Journal of rheumatology. 2013;40(1):66-73.  10. Best A, Terpstra JL, Moor G, Riley B, Norman CD, Glasgow RE. Building knowledge integration systems for evidence-informed decisions. Journal of health organization and management. 2009;23(6):627-41.  11. Boivin A, Marcoux I, Garnon G, Lehoux P, Mays N, Premont MC, et al. Comparing end-of-life practices in different policy contexts: a scoping review. Journal of health services research & policy. 2015;20(2):115-23.  12. Bragge P, Clavisi O, Turner T, Tavender E, Collie A, Gruen RL. The Global Evidence Mapping Initiative: scoping research in broad topic areas. BMC medical research methodology. 2011;11:92.  13. Bravo P, Edwards A, Barr PJ, Scholl I, Elwyn G, McAllister M. Conceptualising patient empowerment: a mixed methods study. BMC health services research. 2015;15:252.  14. Brennan Ramirez LK, Hoehner CM, Brownson RC, Cook R, Orleans CT, Hollander M, et al. Indicators of activity-friendly communities: an evidence-based consensus process. American journal of preventive medicine. 2006;31(6):515-24.  15. Brouwers M, Oliver TK, Crawford J, Ellison P, Evans WK, Gagliardi A, et al. Cancer diagnostic assessment programs: standards for the organization of care in Ontario. Current oncology (Toronto, Ont). 2009;16(6):29-41.  16. Buchan J, Fronteira I, Dussault G. Continuity and change in human resources policies for health: lessons from Brazil. Human resources for health. 2011;9:17.  17. Bush SH, Leonard MM, Agar M, Spiller JA, Hosie A, Wright DK, et al. End-of-life delirium: issues regarding recognition, optimal management, and the role of sedation in the dying phase. Journal of pain and symptom management. 2014;48(2):215-30.  18. Catto AG, Zgaga L, Theodoratou E, Huda T, Nair H, El Arifeen S, et al. An evaluation of oxygen systems for treatment of childhood pneumonia. BMC public health. 2011;11 Suppl 3:S28.  19. Cavazza M, Jommi C. Stakeholders involvement by HTA Organisations: why is so different? Health policy (Amsterdam, Netherlands). 2012;105(2-3):236-45.  20. Choi BC, Li L, Lu Y, Zhang LR, Zhu Y, Pak AW, et al. Bridging the gap between science and policy: an international survey of scientists and policy makers in China and Canada. Implementation science : IS. 2016;11:16.  21. Clark R, Waters E, Armstrong R, Conning R, Allender S, Swinburn B. Evidence and obesity prevention: developing evidence summaries to support decision making. Evidence & Policy: A Journal of Research, Debate and Practice. 2013;9(4):547-56.  22. Clarke D, Duke J, Wuliji T, Smith A, Phuong K, San U. Strengthening health professions regulation in Cambodia: a rapid assessment. Human resources for health. 2016;14:9.  23. Concannon TW, Fuster M, Saunders T, Patel K, Wong JB, Leslie LK, et al. A systematic review of stakeholder engagement in comparative effectiveness and patient-centered outcomes research. Journal of general internal medicine. 2014;29(12):1692-701.  24. Cooray SE, Bhaumik S, Roy A, Devapriam J, Rai R, Alexander R. Intellectual disability and the ICD-11: towards clinical utility? Advances in Mental Health and Intellectual Disabilities. 2015;9(1):3-8.  25. Cottrell E, Whitlock E, Kato E, Uhl S, Belinson S, Chang C, et al. AHRQ Methods for Effective Health Care. Defining the Benefits of Stakeholder Engagement in Systematic Reviews. Rockville (MD): Agency for Healthcare Research and Quality (US); 2014.  26. Crawford C, Boyd C, Jain S, Khorsan R, Jonas W. Rapid Evidence Assessment of the Literature (REAL((c))): streamlining the systematic review process and creating utility for evidence-based health care. BMC research notes. 2015;8:631.  27. Crowley MJ, McCrory DC, Chatterjee R, Gierisch JM, Myers ER, Schmit KM, et al. Prioritization of research addressing antipsychotics for adolescents and young adults with bipolar disorder. Annals of internal medicine. 2014;160(7):492-8.  28. Dahabreh IJ, Trikalinos TA, Balk EM, Wong JB. Recommendations for the Conduct and Reporting of Modeling and Simulation Studies in Health Technology Assessment. Annals of internal medicine. 2016;165(8):575-81.  29. Deverka PA, Lavallee DC, Desai PJ, Esmail LC, Ramsey SD, Veenstra DL, et al. Stakeholder participation in comparative effectiveness research: defining a framework for effective engagement. J Comp Eff Res. 2012;1(2):181-94.  30. DiCenso A, Martin-Misener R, Bryant-Lukosius D, Bourgeault I, Kilpatrick K, Donald F, et al. Advanced practice nursing in Canada: overview of a decision support synthesis. Nursing leadership (Toronto, Ont). 2010;23 Spec No 2010:15-34.  31. Entwistle V, Firnigl D, Ryan M, Francis J, Kinghorn P. Which experiences of health care delivery matter to service users and why? A critical interpretive synthesis and conceptual map. Journal of health services research & policy. 2012;17(2):70-8.  32. Fox A, Chenhall C, Traynor M, Scythes C, Bellman J. Public health nutrition practice in Canada: a situational assessment. Public health nutrition. 2008;11(8):773-81.  33. Freijser L, Naccarella L, McKenzie R, Krishnasamy M. Cancer care coordination: building a platform for the development of care coordinator roles and ongoing evaluation. Australian journal of primary health. 2015;21(2):157-63.  34. Goodman C, Dening T, Gordon AL, Davies SL, Meyer J, Martin FC, et al. Effective health care for older people living and dying in care homes: a realist review. BMC health services research. 2016;16:269.  35. Guise JM, O'Haire C, McPheeters M, Most C, Labrant L, Lee K, et al. A practice-based tool for engaging stakeholders in future research: a synthesis of current practices. Journal of clinical epidemiology. 2013;66(6):666-74.  36. Haigh FA, Scott-Samuel A. Engaging communities to tackle anti-social behaviour: a health impact assessment of a citizens' jury. Public health. 2008;122(11):1191-8.  37. Hatziandreu E, Archontakis F, Daly A. The potential cost savings of greater use of home and hospice-based end of life care in England. RAND Corporation 2008.  38. Hayden JA, Killian L, Zygmunt A, Babineau J, Martin-Misener R, Jensen JL, et al. Methods of a multi-faceted rapid knowledge synthesis project to inform the implementation of a new health service model: Collaborative Emergency Centres. Systematic reviews. 2015;4:7.  39. Haynes RB, Wilczynski NL. Effects of computerized clinical decision support systems on practitioner performance and patient outcomes: methods of a decision-maker-researcher partnership systematic review. Implementation science : IS. 2010;5:12.  40. Higashi H, Khuong TA, Ngo AD, Hill PS. The development of Tobacco Harm Prevention Law in Vietnam: stakeholder tensions over tobacco control legislation in a state owned industry. Substance abuse treatment, prevention, and policy. 2011;6:24.  41. Higginbottom GM, Morgan M, Alexandre M, Chiu Y, Forgeron J, Kocay D, et al. Immigrant women's experiences of maternity-care services in Canada: a systematic review using a narrative synthesis. Systematic reviews. 2015;4:13.  42. Hillier S, Comans T, Sutton M, Amsters D, Kendall M. Development of a participatory process to address fragmented application of outcome measurement for rehabilitation in community settings. Disability and rehabilitation. 2010;32(6):511-20.  43. Horgan D, Jansen M, Leyens L, Lal JA, Sudbrak R, Hackenitz E, et al. An index of barriers for the implementation of personalised medicine and pharmacogenomics in Europe. Public health genomics. 2014;17(5-6):287-98.  44. Jagosh J, Macaulay AC, Pluye P, Salsberg J, Bush PL, Henderson J, et al. Uncovering the benefits of participatory research: implications of a realist review for health research and practice. The Milbank quarterly. 2012;90(2):311-46.  45. Jeon YH, Glasgow NJ, Merlyn T, Sansoni E. Policy options to improve leadership of middle managers in the Australian residential aged care setting: a narrative synthesis. BMC health services research. 2010;10:190.  46. Jeon YH, Sansoni J, Low LF, Chenoweth L, Zapart S, Sansoni E, et al. Recommended measures for the assessment of behavioral disturbances associated with dementia. The American journal of geriatric psychiatry : official journal of the American Association for Geriatric Psychiatry. 2011;19(5):403-15.  47. Kaplan GE, Juhl AL, Gujral IB, Hoaglin-Wagner AL, Gabella BA, McDermott KM. Tools for identifying and prioritizing evidence-based obesity prevention strategies, Colorado. Preventing chronic disease. 2013;10:E106.  48. Keown K, Van Eerd D, Irvin E. Stakeholder engagement opportunities in systematic reviews: knowledge transfer for policy and practice. J Contin Educ Health Prof. 2008;28(2):67-72.  49. Khan Y, Fazli G, Henry B, de Villa E, Tsamis C, Grant M, et al. The evidence base of primary research in public health emergency preparedness: a scoping review and stakeholder consultation. BMC public health. 2015;15:432.  50. Khangura S, Konnyu K, Cushman R, Grimshaw J, Moher D. Evidence summaries: the evolution of a rapid review approach. Systematic reviews. 2012;1:10.    51. Knapp P, Chait A, Pappadopulos E, Crystal S, Jensen PS. Treatment of maladaptive aggression in youth: CERT guidelines I. Engagement, assessment, and management. Pediatrics. 2012;129(6):e1562-76.  52. Konnyu KJ, Kwok E, Skidmore B, Moher D. The effectiveness and safety of emergency department short stay units: a rapid review. Open medicine : a peer-reviewed, independent, open-access journal. 2012;6(1):e10-6.  53. Lavis J, Davies H, Oxman A, Denis JL, Golden-Biddle K, Ferlie E. Towards systematic reviews that inform health care management and policy-making. Journal of health services research & policy. 2005;10 Suppl 1:35-48.  54. Maxwell M, Harris P, Peters S, Thornell M, D'Souza L. A health impact assessment on the construction phase of a major hospital redevelopment. Australian health review : a publication of the Australian Hospital Association. 2008;32(3):509-19.  55. McGinn CA, Gagnon MP, Shaw N, Sicotte C, Mathieu L, Leduc Y, et al. Users' perspectives of key factors to implementing electronic health records in Canada: a Delphi study. BMC medical informatics and decision making. 2012;12:105.  56. McIntosh HM, Calvert J, Macpherson KJ, Thompson L. The Healthcare Improvement Scotland evidence note rapid review process: providing timely, reliable evidence to inform imperative decisions on healthcare. International journal of evidence-based healthcare. 2016;14(2):95-101.  57. McNeill G, Osei-Assibey G, Dick S, Macdiarmid J, Semple S, Reilly J, et al. P32 Using evidence to prioritise areas for public health actions for tackling childhood overweight. Journal of Epidemiology and Community Health. 2010;64(Suppl 1):A46-A.  58. Mindell J, Bowen C, Herriot N, Findlay G, Atkinson S. Institutionalizing health impact assessment in London as a public health tool for increasing synergy between policies in other areas. Public health. 2010;124(2):107-14.  59. Muller L, Flisher A. Standards for the mental health care of people with severe psychiatric disorders in South Africa: Part 2. Methodology and results. South African Psychiatry Review. 2005;8:146-52.  60. Nay R, Koch S. Overcoming restraint use: examining barriers in Australian aged care facilities. Journal of gerontological nursing. 2006;32(1):33-8.  61. Nielsen CP, Funch TM, Kristensen FB. Health technology assessment: research trends and future priorities in Europe. Journal of health services research & policy. 2011;16 Suppl 2:6-15.  62. Oborn E, Barrett M, Racko G. Knowledge translation in healthcare: Incorporating theories of learning and knowledge from the management literature. Journal of health organization and management. 2013;27(4):412-31.  63. O'Brien K, Wilkins A, Zack E, Solomon P. Scoping the field: identifying key research priorities in HIV and rehabilitation. AIDS and behavior. 2010;14(2):448-58.  64. Oliver S, Dickson K. Policy-relevant systematic reviews to strengthen health systems: models and mechanisms to support their production. Evidence & Policy: A Journal of Research, Debate and Practice. 2016;12(2):235-59.  65. Orem JN, Mafigiri DK, Marchal B, Ssengooba F, Macq J, Criel B. Research, evidence and policymaking: the perspectives of policy actors on improving uptake of evidence in health policy development and implementation in Uganda. BMC public health. 2012;12:109.  66. Pakes B. Ethical Analysis in Public Health Practice- Heterogeny, Discensus an the Man-on-the-Clapham Omnibus Toronto: University of Toronto; 2014.  67. Pineault R, Lamarche P, Beaulieu M-D, Haggerty J, Larouche D, Jalhay J-M, et al. Conceptual and methodological challenges in producing research syntheses for decision-and policy-making: an Illustrative case in primary healthcare. Evaluation. 2010;16(2):137-52.  68. Pomey MP, Forest PG, Sanmartin C, Decoster C, Clavel N, Warren E, et al. Toward systematic reviews to understand the determinants of wait time management success to help decision-makers and managers better manage wait times. Implementation science : IS. 2013;8:61.  69. R. Makkar S, Brennan S, Turner T, Williamson A, Redman S, Green S. The development of SAGE: A tool to evaluate how policymakers’ engage with and use research in health policymaking. Research Evaluation. 2016;25(3):315-28.  70. Rikard-Bell G, Waters E, Ward J. Evidence-based clinical policy: case report of a reproducible process to encourage understanding and evaluation of evidence. Internal medicine journal. 2006;36(7):452-7.  71. Rowan M, Poole N, Shea B, Gone JP, Mykota D, Farag M, et al. Cultural interventions to treat addictions in Indigenous populations: findings from a scoping study. Substance abuse treatment, prevention, and policy. 2014;9:34.  72. Rycroft-Malone J, Burton CR, Williams L, Edwards S, Fisher D, Hall B, et al. Health Services and Delivery Research. Improving skills and care standards in the support workforce for older people: a realist synthesis of workforce development interventions. Southampton (UK): NIHR Journals Library 2016.  73. Saul JE, Willis CD, Bitz J, Best A. A time-responsive tool for informing policy making: rapid realist review. Implementation science : IS. 2013;8:103.  74. Schoo AM, R AB, Ridoutt L, Santos T. Workload capacity measures for estimating allied health staffing requirements. Australian health review : a publication of the Australian Hospital Association. 2008;32(3):548-58.  75. Sidibe S, Pack AP, Tolley EE, Ryan E, Mackenzie C, Bockh E, et al. Communicating about microbicides with women in mind: tailoring messages for specific audiences. Journal of the International AIDS Society. 2014;17(3 Suppl 2):19151.  76. Teerawattananon Y, Kingkaew P, Koopitakkajorn T, Youngkong S, Tritasavit N, Srisuwan P, et al. Development of a Health Screening Package Under the Universal Health Coverage: The Role of Health Technology Assessment. Health economics. 2016;25 Suppl 1:162-78.  77. Thariani R, Wong W, Carlson JJ, Garrison L, Ramsey S, Deverka PA, et al. Prioritization in comparative effectiveness research: the CANCERGEN Experience. Medical care. 2012;50(5):388-93.  78. Verhoef LM, Van de Belt TH, Engelen LJ, Schoonhoven L, Kool RB. Social media and rating sites as tools to understanding quality of care: a scoping review. Journal of medical Internet research. 2014;16(2):e56.  79. Whitaker R, Hendry M, Aslam R, Booth A, Carter B, Charles JM, et al. Intervention Now to Eliminate Repeat Unintended Pregnancy in Teenagers (INTERUPT): a systematic review of intervention effectiveness and cost-effectiveness, and qualitative and realist synthesis of implementation factors and user engagement. Health technology assessment (Winchester, England). 2016;20(16):1-214.  80. Wilhelm B, Waddell L, Greig J, Rajic A, Houde A, McEwen SA. A scoping review of the evidence for public health risks of three emerging potentially zoonotic viruses: hepatitis E virus, norovirus, and rotavirus. Preventive veterinary medicine. 2015;119(1-2):61-79.  81. Willis CD, Saul JE, Bitz J, Pompu K, Best A, Jackson B. Improving organizational capacity to address health literacy in public health: a rapid realist review. Public health. 2014;128(6):515-24.  82. Wilson MG, Beland F, Julien D, Gauvin L, Guindon GE, Roy D, et al. Interventions for preventing, delaying the onset, or decreasing the burden of frailty: an overview of systematic reviews. Systematic reviews. 2015;4:128.  83. Wiysonge CS, Ngcobo NJ, Jeena PM, Madhi SA, Schoub BD, Hawkridge A, et al. Advances in childhood immunisation in South Africa: where to now? Programme managers' views and evidence from systematic reviews. BMC public health. 2012;12:578.  84. Worthington C, O'Brien K, Zack E, McKee E, Oliver B. Enhancing labour force participation for people living with HIV: a multi-perspective summary of the research evidence. AIDS and behavior. 2012;16(1):231-43. |
| **Companion documents (n=7)** |
| 85. Carter N, Martin-Misener R, Kilpatrick K, Kaasalainen S, Donald F, Bryant-Lukosius D, et al. The role of nursing leadership in integrating clinical nurse specialists and nurse practitioners in healthcare delivery in Canada. Nursing leadership (Toronto, Ont). 2010;23 Spec No 2010:167-85.  86. Leonard MM, Agar M, Spiller JA, Davis B, Mohamad MM, Meagher DJ, et al. Delirium diagnostic and classification challenges in palliative care: subsyndromal delirium, comorbid delirium-dementia, and psychomotor subtypes. Journal of pain and symptom management. 2014;48(2):199-214.  87. Nieuwlaat R, Connolly SJ, Mackay JA, Weise-Kelly L, Navarro T, Wilczynski NL, et al. Computerized clinical decision support systems for therapeutic drug monitoring and dosing: a decision-maker-researcher partnership systematic review. Implementation science : IS. 2011;6:90.  88. Roshanov PS, You JJ, Dhaliwal J, Koff D, Mackay JA, Weise-Kelly L, et al. Can computerized clinical decision support systems improve practitioners' diagnostic test ordering behavior? A decision-maker-researcher partnership systematic review. Implementation science : IS. 2011;6:88.  89. Sahota N, Lloyd R, Ramakrishna A, Mackay JA, Prorok JC, Weise-Kelly L, et al. Computerized clinical decision support systems for acute care management: a decision-maker-researcher partnership systematic review of effects on process of care and patient outcomes. Implementation science : IS. 2011;6:91.  90. Scotto Rosato N, Correll CU, Pappadopulos E, Chait A, Crystal S, Jensen PS. Treatment of maladaptive aggression in youth: CERT guidelines II. Treatments and ongoing management.  91. Souza NM, Sebaldt RJ, Mackay JA, Prorok JC, Weise-Kelly L, Navarro T, et al. Computerized clinical decision support systems for primary preventive care: a decision-maker-researcher partnership systematic review of effects on process of care and patient outcomes. Implementation science : IS. 2011;6:87. |

# **Appendix 3.** Definitions

| **Term** | **Definition** |
| --- | --- |
| Advisory group | A small multi-stakeholder group that advises on various stages of a research project starting from planning to dissemination and implementation. The advisory group members do not conduct the research themselves, but rather provide recommendations on the design and method.  *Synonyms: Advisory panel, reference group, advisory committee* |
| Expert panel | A larger multi-stakeholder group established to provide specialized input and opinion by means of Delphi or nominal group technique or other information-gathering approaches. The expert panel can be engaged at various stages of a review to solicit feedback and establish consensus on various courses of action and make recommendations. |
| Key informant or stakeholder | Individuals with specialized knowledge and informed perspectives on the research area who can add valuable sources of information to the research project. |
| Principal knowledge user | Stakeholder that either requested or commissioned the research project. |
| Steering group | A small multi-stakeholder group that provide strategic decisions at key stages during the course of a research project. The steering group members do not conduct the research themselves, but rather make key decisions during the research process. While an advisor group provides advice on the research project, a steering group is responsible for making concrete decisions.  *Synonym: Steering committee* |
| Team member | A stakeholder involved in the conduct of the research as a part of the research team.  *Synonym: Project team member* |
| Working group | A small multidisciplinary stakeholder group established to perform a specific task of a research project.  *Synonyms: Task force, project team* |
